# Colpo-Myotomy

**Published:** 1886-08

**Authors:** J. W. Carhart

**Affiliations:** Lampasas, Texas


### COLPO-MYOTOMY.

*By J. W. Carhart, M. D., Lampasas, Texas.*

(For Daniel's Texas Medical Journal.)

MYOMA of the uterus is an hyperplasia of some portion of the uterine tissue. If the hyperplasia be of the connective tissue we call the tumor a fibro-myoma. If it be of the muscular tissue, it is classified as cellular. If of the glands of the organ, it is classified as glandular. Authors differ, however, in their classification, and as a result an almost indefinite number of names have been applied to these growths.

It is not my purpose in this paper to discuss the various myomatous growths to which the uterus is subject; but simply as preliminary to an account of an operation, to present some features of uterine polypi; more especially since the literature on the subject is not as abundant as on some other forms of uterine disease. It is noticeable that Galen, Hippocrates, Celsus, and other ancient writers, make no mention of uterine polypi whatever; and, to my surprise, I find, on examining several modern authors on diseases of women, that the subject is omitted altogether, or else only incidentally alluded to.

A submucous myoma cannot develop into the firmer uterine parenchyma, and therefore must distend into the uterine cavity. It may remain there as a polypus sessile on a broad base, or it may proliferate into the uterine cavity, attached to the parenchyma by a narrow pedicle. In this form it is often delivered into the vagina. The pedicle is usually attached to one wall of the uterus, and by contractions of the organ and the dragging force exerted upon the pedicle, the point of attachment is usually moved downwards until it reaches the cervix. In this condition it may vary in size, often no larger than a marble, and occasionally of such bulk as to completely fill the vagina. It is covered with mucous membrane, and within, consists of connective tissue in a state of hypergenesis. The tissue of the pedicle is usually firm and persistent, and is traversed by abundant nutrient blood vessels. Accounts of hollow polypoid uterine growths are to be found in medical literature.

Uterine polypoid growths are subject to morbid changes; for example,—calcareous, fatty, and malignant. Occasionally we find the pedicles of a polypus, delivered into the vagina, so constricted by a rigid and contracted os as to produce congestive edœma, which may result in gangrene. Such condition of the tumor may follow also injury from coitus or some other cause, when sloughing of the whole tumor may take place; and possibly a spontaneous cure; or, more likely, septicæmia and death.

The diagnosis of a polypus, already delivered into the vagina, and the location of its pedicle attachment, are not difficult, provided the mass is not so large that the fingers cannot be passed up to the cervix uteri. In that case, a satisfactory diagnosis might be difficult, and mistakes have been made by eminent men.

For the removal of pediculated polypi, lying in the vagina *laparo-myotomy* is not to be thought of. *Colpo-myotomy*, or the attack of the tumor from below, may be effected in a variety of ways, of which the following are principal, viz:

Excision;
Torsion and traction;
Ecrasement;
The galvano-caustic wire;
The spoon saw.

The above is Thomas' classification of methods. He further says: "Should the pedicle be within reach of knife or scissors, it may be divided. * * Should the growths be small * * they may be scraped from their attachment by a large steel curette."

Since serious hæmorrhage may ensue from cutting with knife or scissors a mass of tissue traversed by such an abundance of blood vessels as is the pedicle to one of these tumors, that method of operation is to be carefully considered before undertaken. Should that method be chosen, the tumor must be removed with dispatch, in order to allow of the speedy application of hæmostatics.

Few practitioners in provincial districts have an armamentarium so thoroughly equipped as to be able to elect a method fully suited to each case. Ways and means are frequently to be devised, and methods are often resorted to, in various operations, which are not laid down in the books, and not infrequently with complete success. Often the methods of the books have to be combined for lack of instruments, or to meet peculiarities as they arise, and secure the best results.

These facts are illustrated, to some extent, in the following case:

Mrs. J., aged 30 years, white, a native of Texas, married ten years, the mother of four children, the youngest sixteen months old; is slight and delicate in form, of nervous temperament, and rather delicate in health. She complained of metrorrhagia whilst I was treating her husband for typhoid fever; said her menses had ceased three months before, and she frequently experienced dragging pains, but declined treatment, saying she thought it would soon "pass off." During my absence from town, however, on November 13, 1885, she became alarmed at the amount of hæmorrhage, and finding me absent, called another physician, who did not propose an examination, but prescribed ergot and black haw. On my return, on the morning of the 14th, I answered the call, supposing I should find her husband worse. I found Mrs. J. flooding badly, although not so bad, she said, as the night before. Supposing her to be pregnant, and that a miscarriage was threatened, I proposed an examination, which was readily consented to, and on the introduction of the fingers I found a mass lying in the vagina, which o i the instant I took to be the fœtus and membranes. She said she did not think she was "in a family way." I discovered that the feel of the mass was not that of a fœtus and membranes, but more that of a placenta. Passing my index finger up to the cervix uteri, I felt what seemed to me to be the cord, and was inclined to think it a case of placenta previa. This thought was at once dispelled by the contracted condition of the os, which, however, admitted the finger far enouhg to feel the attachment of the pedicle to the side of the cervix. I was then certain that I had to deal with a polypus, the size of a man's fist, with a pedicle not larger than one's little finger.

Not being provided with an ecrasseur of any sort, my first attempt was to remove it by torsion and traction. I failed in this, as it was too slippery for my fingers, and I had no vulsellum or spoon forceps with which to grasp it. I might have reached the pedicle, perhaps, with long, curved, blunt scissors, used in "Emmet's operation," but I feared the laceration of adjacent parts, and still more the hæmorrhage that might follow before I could remove the mass from the vagina and apply hot water or a hæmostatic tamponade. I therefore decided to dissect away the tumor and scrape off the detritus of the pedicle from the walls of the cervix with a sharp steel curette, which I did, partly cutting the tumor with blunt scissors, and partly tearing it with my fingers, keeping the hæmorrhage thoroughly under control by occasional injections of water at 112° F. The patient was in a dorsal decubitus, with feet drawn up and knees separated. No anæsthetic was given, and no one present to render me any assistance.

The curette removed completely the tenacious tissue of the attachment, which gave free hæmorrhage, but which was promptly controlled by the use of hot water. Plenty of time was taken, as the patient suffered but little pain and but slight exhaustion.

A few doses of bromide of potassium and chloral-hydrate were given during the night; and the following morning injections of warm carbolized water were ordered and continued for several days. The patient made a quick and uninterrupted recovery; and informs me at this writing that she has menstruated regularly since, with no sign of metrorrhagia.

---

*Dr. F. E. Daniel* of Austin has been tendered the appointment of Secretary of the Texas State Medical Association vice Dr. W. J. Burt, deceased.

---

It is supposed that on the retirement of General Murray this month, the Medical officers of the U. S. Army will turn their Baxter the Surgeon General.

